# Liver damage in HIV+HBV co-infected patients determined by transient elastography

**DOI:** 10.1186/1471-2334-14-S4-P18

**Published:** 2014-05-29

**Authors:** Elena Dumea, Adrian Streinu-Cercel, Sorin Rugină, Lucian Cristian Petcu, Zizi Niculescu, Alina Doina Nicoară, Simona Claudia Cambrea

**Affiliations:** 1 Ovidius University, Constanța, Romania; 2 Clinical Hospital of Infectious Diseases, Constanța, Romania; 3 Carol Davila University of Medicine and Pharmacy, Bucharest, Romania

## 

In our country the prevalence of HIV-HBV co-infection in young infected patients, between 1985-1990, transmitted through nosocomial or vertical path is approximately 40%.

We followed the prevalence and risk factors associated with liver damage in HIV+HBV infected patients. Longitudinal evaluation of liver fibrosis was carried out in the patients included in the study group, by transient elastography (TE). Several studies using non-invasive methods for the assessment of fibrosis have been performed in HIV infected patients, and in patients co-infected with hepatitis virus B, although up to now these methods have not been validated for this segment of the population. For statistical analysis, the TE results were designated to different stages of fibrosis in accordance with the previous recommendations. The predefined cut-off values were: F0-F1≤7.1 kPa, F2-F3>7.1 and ≤12.5 kPa and for cirrhosis (corresponding to F4) >12.5kPa.

We included in the study 71 patients co-infected with HIV and hepatitis B. 71.85% of patients had minimal liver damage, 18.30% of them had moderate to severe fibrosis, 9.85% were F4. Patients were divided according to CD4 count into three groups: CD4 (0-200)/cmm, [200-500)/cmm, and >500 cells/cmm. By applying the ANOVA test we found significant differences between the 3 groups (p=0.037<α=0.05, F=3.472) and reading the Bonferroni table shows that there are significant differences between the values of FibroScan only in patients coinfected with HIV+HBV with CD4 appropriate intervals (0 - 200] and (>500) [cells/cmm], p=0.033<α=0.05, confidence interval (CI) 95% (0139, 4572). We didn`t find significant differences regarding patients’ liver disease between groups with HIV-RNA viral load <400 copies/mL compared to those with levels >400 copies/mL. For assessing the role of hepatitis virus B in liver disease severity in co-infected patients, patients were divided into two groups: HBV-DNA level≤ 2,000 IU/mL and >2,000 IU/mL. Since p=0.006<α=0.05, there are significant differences between the mean values of FibroScan in the two groups, HIV+HBV patients with HBV-DNA (0-2,000 IU/mL and >2,000 IU/mL), 95%CI (-4.642,-0.788).

We confirm the role of HIV-induced immunosuppression in liver disease progression. As well we confirm the presence of more severe liver disease linked to hepatitis virus B replication.

